# Extracellular α-synuclein levels are regulated by neuronal activity

**DOI:** 10.1186/s13024-018-0241-0

**Published:** 2018-02-22

**Authors:** Kaoru Yamada, Takeshi Iwatsubo

**Affiliations:** Department of Neuropathology, Graduate School of Medicine, The University of Tokyo, 7-3-1, Hongo, Bunkyo-ku, Tokyo, 113-0033 Japan

**Keywords:** α-synuclein, Parkinson’s disease, Propagation, Neuronal activity

## Abstract

**Background:**

α-Synuclein is a presynaptic protein abundant in the cytoplasmic compartment of neurons, whereas its presence in the extracellular space has also been observed under physiological conditions. Extracellular α-synuclein has pathological significance, exhibiting cellular toxicity and impairment of synaptic transmission. Notably, misfolded α-synuclein drives the cell-to-cell propagation of pathology via the extracellular space. However, the primary mechanism that regulates the extracellular levels of α-synuclein remains to be determined.

**Methods:**

Using several mechanistically distinct methods to modulate neuronal/synaptic activities in primary neuronal culture and in vivo microdialysis, we examined the involvement of neuronal/synaptic activities on α-synuclein release.

**Results:**

We demonstrate here that physiological release of endogenous α-synuclein highly depends on intrinsic neuronal activities. Elevating neuronal activity rapidly increased, while blocking activity decreased, α-synuclein release. In vivo microdialysis experiments in freely moving mice revealed that ~ 70% of extracellular α-synuclein arises from neuronal activity-dependent pathway. Selective modulation of glutamatergic neurotransmission altered extracellular α-synuclein levels, implicating this specific neuronal network in the mechanism of activity-dependent release of α-synuclein. While neuronal activity tightly regulated α-synuclein release, elevated synaptic vesicle exocytosis per se was capable to elicit α-synuclein release. We also found that extracellular α-synuclein exists as high molecular weight species.

**Conclusions:**

The present study uncovers a novel regulatory pathway associated with α-synuclein release, whose dysregulation might affect various pathological actions of extracellular α-synuclein including its trans-synaptic propagation.

## Background

The pathological aggregation of α-synuclein characterizes Parkinson’s disease, dementia with Lewy bodies and multiple system atrophy collectively referred to as α-synucleinopathies [1, 2]. While α-synuclein aggregates intracellularly, accumulating evidence suggests that extracellular α-synuclein also has pathological significance [3]: studies have demonstrated that aggregated forms of extracellular α-synuclein trigger trans-cellular spreading of α-synuclein pathology [4–7]. Extracellular α-synuclein oligomers impairs long-term potentiation (LTP), alters the glutamatergic synaptic transmission and α3-Na^+^/K^+^-ATPase activity [8, 9], and also activates inflammatory response in microglia [10]. Secreted monomeric α-synuclein has been also shown to exhibit toxicity to cells [11], perturb calcium homeostasis [12] and induce fragmentation of lipid rafts [13].

α-Synuclein is present in media of various cells overexpressing α-synuclein and peripheral neurons [6, 11, 14, 15], brain interstitial fluid (ISF) [16] and also in cerebrospinal fluid [17, 18], suggesting that its release occurs independently of cell death. Now the phenomenon of α-synculein release has been well documented, whereas the underlying mechanism that regulates α-synuclein release has remained poorly investigated. To better understand the physiological release of α-synuclein, we have examined the relationship between neuronal activity and α-synuclein release both in cultured neurons and living mice.

## Results

### Neuronal activity drives de novo release of α-synuclein from neurons

We hypothesized that neuronal activity regulates physiological release of α-synuclein. Due to the artificial influences of over-expressed α-synuclein on the secretory mechanism [19] as well as basal synaptic transmission [20, 21], we sought to measure release of endogenous α-synuclein from cultured neurons.

**Fig. 1:**
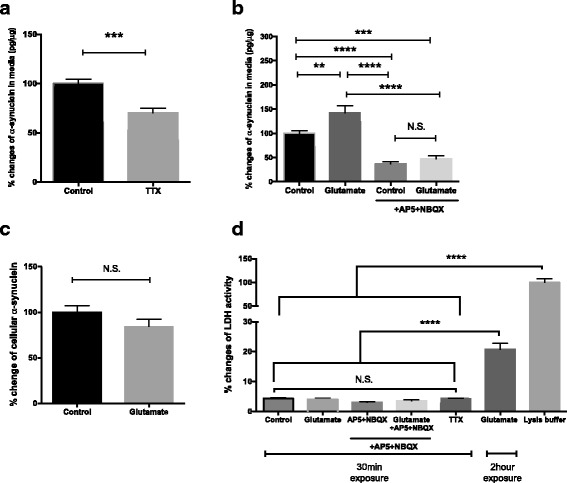
Modulating neuronal activity alters α-synuclein release from neurons. **a** TTX (1 μM, 30 min) decreased α-synuclein in the media. *N* = 12–16. **b** Glutamate (10 μM, 30 min) increased α-synuclein in the media, which was blocked by 50 μM AP5 and 10 μM NBQX. *N* = 8–20. **c** Cellular α-synuclein levels were not altered by glutamate. *N* = 8. **d** LDH activities in the media after indicated pharmacological treatments. N = 8–28. mean ± SEM, **p < 0.01, ***p < 0.001, ****p < 0.0001 using unpaired t test (**a**, **c**) or one-way ANOVA (**b**, **d**)

Consistent with the hypothesis, we found that tetrodotoxin (TTX), a sodium channel blocker that inhibits the generation of action potential, significantly decreased α-synuclein levels released in media (Fig. 1a).

The expression of α-synuclein at the synapses of excitatory neurons [22, 23] led us to examine the involvement of excitatory neurotransmission. Thirty minutes exposure of glutamate increased extracellular α-synuclein levels. Pre-incubation with a specific NMDA receptor antagonist, AP5, and an AMPA receptor antagonist, NBQX, abolished both basal and glutamate-evoked α-synuclein release (Fig. 1b). The increase in α-synuclein release by glutamate was not associated with changes in cellular α-synuclein levels (Fig. 1c). While prolonged exposure of glutamate at higher dose (2.5 h treatment of 100 μM glutamate) increased the non-specific release of lactate dehydrogenase (LDH) in the media likely due to excitotoxicity, such increase was not observed in 30 min exposure of 20 μM glutamate and other treatment groups (Fig. 1d). This demonstrates that neuronal activity influenced extracellular α-synuclein levels by modulating the release, not by augmenting excitotoxicity or altering its expression level.

### Neuronal activity alters the steady-state levels of extracellular α-synuclein in vivo

**Fig. 2:**
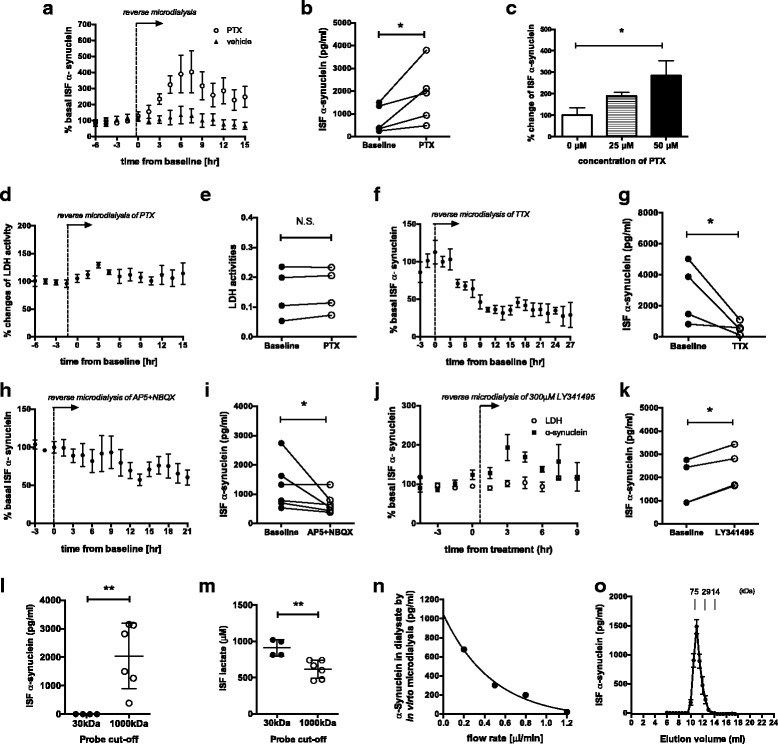
ISF α-synuclein is present as high molecular weight species and its steady-state levels are dynamically regulated by neuronal activity. **a** Picrotoxin (PTX, 50 μM) increased ISF α-synuclein compared to vehicle treatment. *N* = 4–5. **b** ISF α-synuclein concentrations were increased by 50 μM PTX from baseline. *N* = 5. **c** The mean ISF α-synuclein levels after 25 μM or 50 μM PTX or vehicle (0 μM) infusion were elevated dose dependently N = 4–7. **d** % ISF LDH activities did not alter by 50 μM PTX. *N* = 4. **e** ISF LDH activities did not change by 50 μM PTX from baseline. N = 4. **f** Tetrodotoxin (5 μM, TTX) decreased ISF α-synuclein N = 4. **g** ISF α-synuclein concentrations were decreased by TTX from baseline. N = 4. **h** 50 μM AP5 and 10 μM NBQX decreased ISF α-synuclein. *N* = 6. **i** ISF α-synuclein concentrations were decreased by AP5 and NBQX from baseline. N = 6. **j** 300 μM LY341495 increased ISF α-synuclein without changing LDH activities N = 4. **k** ISF α-synuclein concentrations were increased by LY341495 from baseline. N = 4. The effect of drug treatment was assessed between baseline α-synuclein levels and mean α-synuclein levels in the entire treatment period. **l** Absolute concentration of ISF α-synuclein collected at 1.0 μl/min through either 30 kDa membranes or 1000 kDa membranes. N = 4–6. **m** Absolute concentration of ISF lactate collected at 1.0 μl/min through either 30 kDa membranes or 1000 kDa membranes. N = 4–6. **n** α-Synuclein concentrations in dialysate collected at the different flow rate by in vitro microdialysis with 30 kDa membranes. **o** ISF samples obtained from wild-type mice through 1000 kDa membranes were separated by a size exclusion chromatography using a Superdex75 column. α-Synuclein levels in eluted fractions were quantified by α-synuclein ELISA (N = 4). Mean ± SEM, *p < 0.05, **p < 0.01, using one-way ANOVA (**c**) or using ratio paired t test (**b**, **e**, **g**, **i**, **k**) or unpaired t test (**l**, **m**)

We reasoned if neuronal activity is a major regulator of α-synuclein release, altering activity would also change its steady state levels in ISF. To test this in the context of intact neuronal network, we performed in vivo microdialysis that allows the measurement of real-time changes in ISF endogenous α-synuclein on an hourly basis in freely moving mice. Following the collection of basal α-synuclein, picrotoxin (PTX), a GABA_A_ receptor antagonist, was locally and continuously administered to hippocampus through the microdialysis probes with 1000 kDa cut-off membranes by reverse microdialysis to stimulate neurons. PTX significantly enhanced ISF α-synuclein levels (Fig. 2a, b) in a dose dependent manner, suggesting that the degree of neuronal activation determines its levels (Fig. 2c). Consistent with the report that low dose of PTX only produces occasional spikes and does not cause any excitotoxicity [24], PTX did not increase ISF LDH activities (Fig. 2d, e), while the same dose significantly elevated ISF α-synuclein levels.

In contrast to PTX, inhibiting neuronal activity by TTX significantly decreased ISF α-synuclein levels by a maximum of ~ 70% (Fig. 2f, g). TTX reduced ISF α-synuclein not only in hippocampus but also in striatum (data not shown). This striking reduction in ISF α-synuclein levels suggests that the majority of extracellular α-synuclein arises from neuronal activity-dependent mechanisms.

Blocking glutamate receptors by AP5 and NBQX also significantly decreased ISF α-synuclein levels (Fig. 2h, i). Metabotropic glutamate receptors 2/3 negatively regulate glutamate release at the pre-synaptic terminal [24]. Thus, its inhibition enhances glutamate release. Infusion of a highly specific metabotropic receptor 2/3 antagonist, LY341495 slightly but significantly elevated ISF α-synuclein levels without changing LDH activities (Fig. 2j, k).

In order to examine the molecular weight size of ISF α-synuclein, we performed in vivo microdialysis using probes with 30 kDa cut-off membrane, which will predominantly collect monomeric α-synuclein (~ 14 kDa) and possibly a dimeric form (~ 28 kDa). Surprisingly, α-synculein was not detected in ISF collected through 30 kDa membranes although ISF lactate levels were not decreased with 30 kDa compared to 1000 kDa membranes (Fig. 2l, m). Recombinant α-synuclein was detectable by in vitro microdialysis with 30 kDa membranes in a flow rate-dependent manner as indicated by an inverse relationship between concentration and flow rate [25], suggesting that at least monomeric α-synuclein can pass through 30 kDa membranes (Fig. 2n). To further verify whether ISF α-synculein exists in a form larger than 30 kDa, we separated ISF samples collected through 1000 kDa cut-off membranes by a size-exclusion chromatography and measured the α-synuclein levels in individual fractions. ISF α-synuclein was eluted as a single sharp peak with an apparent molecular weight of 60 kDa (Fig. 2o). Although it is uncertain whether this represents α-synuclein oligomer or α-synuclein monomer bound to other proteins, it suggests that α-synuclein forms a high molecular weight complex in ISF.

### Synaptic vesicle exocytosis per se is sufficient to enhance α-synuclein release

Collectively, the results suggest that neuronal activity is one mechanism regulating α-synuclein release. Glutamate activates post-synaptic receptors, depolarizes neurons and increases synaptic vesicle exocytosis. To further define which cellular process is associated with α-synuclein release, we performed pharmacological experiments in neurons.

**Fig. 3:**
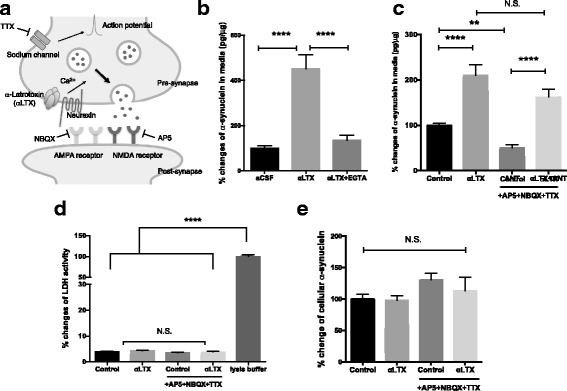
Synaptic vesicle exocytosis is associated with α-synuclein release. **a** Schematic illustration of target molecules for the compounds used in the experiment. **b** α-Latrotoxin (αLTX, 0.5 nM) increased α-synuclein release, which was blocked by 4 mM EGTA. *N* = 8. **c** αLTX significantly increased α-synuclein release in the presence or absence of 1 μM TTX, 50 μM AP5 and 10 μM NBQX. *N* = 16–32. **d** LDH activities in the media were not altered by indicated pharmacological treatments. N = 16–24. **e** Cellular α-synuclein levels were not altered by indicate pharmacological treatments. N = 8. mean ± SEM, ***p < 0.001, ****p < 0.0001 using one-way ANOVA

α-Latrotoxin (αLTX) binds to neurexin receptors on the presynaptic terminals and enhanced synaptic vesicles release through calcium influx independently of pre-synaptic depolarization [26, 27] (Fig. 3a). αLTX increased α-synuclein release, which was blocked in the presence of EGTA (Fig. 3b). Next, to separately assess the effects of synaptic vesicle exocytosis and neuronal activity on α-synuclein release, we treated neurons with αLTX in the presence of TTX, AP5 and NBQX to enhance synaptic vesicle release while simultaneously blocking depolarization and post-synaptic glutamate receptor activation. The inhibitor mixture containing TTX, AP5 and NBQX in the absence of αLTX significantly decreased α-synuclein release. Interestingly, αLTX still enhanced α-synuclein release in the presence of the inhibitor mixture (Fig. 3c), with no changes in LDH activities and cellular α-synuclein levels (Fig. 3d, e). This suggests that pre-synaptic vesicle exocytosis is a critical downstream event associated with α-synuclein release and post-synaptic receptor activation is not the prerequisite.

## Discussion

Prior studies overexpressing α-synuclein have reported multiple pathways that influence α-synuclein release such as proteasomal stress, lysosomal dysfunction, mitochondrial dysfunction [14, 28], and sulfonylurea receptor 1 mediated mechanism [23]. While the studies have shown that cells under external stress release more α-synuclein, the intrinsic mechanism that mediates endogenous α-synuclein release from healthy neurons in brain have not been empirically investigated to date. In the present study, using several mechanistically distinct methods to modulate neuronal activity, we provided substantial evidence that de novo release of endogenous α-synuclein highly depends on neuronal activity. More importantly, by measuring its real-time changes in freely moving wild-type mice, we also demonstrated that neuronal activity impacts the steady-state levels of extracellular α-synuclein in vivo.

Previous studies have suggested that secreted soluble α-synuclein exerts multiple effects. Intriguingly, many of its reported actions are associated with perturbation in neuronal/synaptic function at various levels, such as partitioning of voltage-gated calcium channels [29] or impairment in LTP [8] or alteration in synaptic activity [12] or increase in the susceptibility to depolarization by interacting with α3-Na^+^/K^+^-ATPase [9]. Thus, activity-dependent release of α-synuclein may participate in a dynamic feedback mechanism on neuronal/synaptic function.

Extracellular α-synuclein levels appear to highly rely on intrinsic neuronal activity compared to other secretory proteins released via an activity-dependent manner. For example, TTX only suppressed ISF Aβ levels by ~ 30% despite its complete inhibition of action potentials, suggesting that remaining ~ 70% pool of Aβ release arises from activity-independent mechanisms [27].

The steady-state levels of extracellular α-synuclein are maintained based on the balance between release and clearance. The rapid decrease in ISF α-synuclein by TTX also unveils that α-synuclein is normally cleared rapidly from ISF. Such clearance might be mediated by extracellular protease such as neurosin, which has been shown to degrade α-synuclein in culture media [30]. This is in sharp contrast to tau protein, which has a longer half-life in ISF, despite the common regulatory mechanism of release in an activity dependent manner [24, 31].

Our data suggests the link between synaptic activity and α-synuclein release; nonetheless this is not necessarily equated to its release via synaptic vesicles. Alternatively, it is certainly possible that synaptic activity drives other associated secretory process directly responsible for α-synuclein release.

α-Synuclein release has been shown to occur via an exosome-mediated pathway [5, 11]. Although detection of α-synuclein directly by ELISA in this study suggested the presence of α-synuclein not embedded in extracellular vesicles, it is unknown whether exosome-mediated release also occurs. Currently, the molecular mechanisms for exosome-independent α-synuclein release remains largely unknown and should be explored in the future studies.

Although the current study focuses on the physiological release of α-synuclein, it should further be investigated whether activity-dependent release promotes transmission of α-synuclein pathology. Although it is uncertain that α-synuclein forms oligomers in ISF, our study suggests that extracellular α-synuclein exists as a high molecular weight complex. Given that α-synuclein is capable of oligomerizing in the extracellular space [6], it is tempting to speculate that when local concentration of extracellular α-synuclein at synapses is abnormally elevated by neuronal activation, it may culminate in the chronic accumulation of oligomeric α-synuclein, which could ultimately facilitate the trans-synaptic spreading of α-synuclein pathology. Similar concept has been proposed for the cell-to-cell propagation of tau pathology [32]. From this perspective, the present microdialysis technique with 1000-kDa cut-off membranes might serve as a valuable tool to measure extracellular pathogenic α-synuclein and directly test the efficacy of potential pharmacological treatments in vivo.

## Conclusions

We have demonstrated that neuronal/synaptic activity regulates α-synuclein release in vivo. Trans-synaptic spreading of pathology through neuronal network has been only reported in a certain class of pathogenic cytoplasmic proteins such as α-synuclein and tau. Of note, tau release is also dynamically regulated by synaptic activity [24, 33]. Our findings on the activity-dependent release of α-synuclein may underscore the common mechanistic link between trans-synaptic propagation and the synaptic activities in different types of neurodegenerative disorders.

## Methods

### Compounds

α-Latrotoxin and TTX were purchased from Alamone Labs and Wako Pure Chemical Industries respectively. Glutamate and picrotoxin were purchased from Sigma-Aldrich. D-AP5, NBQX and LY341495 were purchased from Abcam.

### Culture of mouse primary neurons and measurements of α-synculein release from primary neurons

Primary cortical neurons were prepared from embryonic day 14 C57B6/J mouse embryos and plated on dishes coated with poly-D-lysine. Neurons were maintained in Neurobasal medium (Life technologies) containing 1% Glutamax I (Life technologies), 2% B-27 supplement (Life technologies), penicillin, streptomycin and cytosine arabinofuranoside to restrict proliferation of non-neuronal cells. Neurons cultured 14 days in vitro were rinsed once with DPBS and then incubated with Neurobasal phenol red minus medium or artificial CSF (aCSF; 1.3 mM CaCl_2_, 1.2 mM MgSO_4_, 3 mM KCl, 0.4 mM KH_2_PO_4_, 25 mM NaHCO_3_, and 122 mM NaCl, pH 7.4) containing 1% Glutamax I, 2% B-27 supplement with or without indicated chemicals for 30 min at 37 °C (treatment period). To maximize the inhibitory effects of TTX, AP5 and NBQX on the neuronal activity, these inhibitors were added both in treatment period and pre-incubation period (for 2 h at 37 °C). At the end of the experiments, collected media was centrifuged to remove cell debris and the supernatant was used for the further analysis. Neurons were lysed with RIPA (150 mM NaCl, 50 mM Tris, 0.5% deoxycholic acid, 1% Triton X-100, and 0.5% SDS, 25 mM EDTA, pH 8.0) supplemented with Complete (Roche) and PhosSTOP (Roche). α-Synuclein levels were normalized by cellular protein levels determined with BCA assay. To minimize inter-experiment variations, α-synuclein levels/total protein were further normalized by untreated control samples in the same experiment.

### In vivo microdialysis

In vivo microdialysis using 1000 kDa cut-off Atmos microdialysis probes (Eicom microdialysis) was performed with slight modifications from the previously described method [24, 25]. A guide cannula was stereotaxically implanted in the hippocampus (bregma − 3.1 mm, 2.5 mm lateral to midline, 1.2 mm below dura at a 12.5° angle) or the striatum (bregma + 0.5 mm, 1.8 mm lateral to midline, 1.4 mm below dura at a 0° angle) under anesthesia and cemented. A Syringe pump (KDS-101, KD scientific) and a roller pump (ERP-10, Eicom microdialysis) were operated simultaneously for a push-pull microdialysis [34]. As a perfusion buffer, 4% BSA solution in aCSF was made fresh on the day and filtered through a 0.1 μm membrane. The probe was inserted through a guide cannula and perfused at 10 μl/min for 1 h and then ISF was collected at 1.0 μl/min by refrigerated fraction collectors (Univentor 820 microsampler, Univentor). Microdialysis with 30 kDa cut-off membranes (BR-2 probes, BASi) was operated only by a syringe pump. To ensure the stable collection of α-synuclein, the first 9 h fractions were excluded from analysis. The inclusion of chemicals in the perfusion buffer allows them to diffuse out to ISF. This technique is called reverse microdialysis. Reverse microdialysis was performed in hippocampus unless specifically mentioned. To infuse chemicals via reverse microdialysis, a normal perfusion buffer was switched to a perfusion buffer containing various chemicals. As a vehicle control for picrotoxin, a perfusion buffer containing 0.05% DMSO was administered. To assess the maximal effect of TTX or AP5 + NBQX, the mean α-synuclein or LDH levels during the final 3 h of treatment were compared.

### In vitro microdialysis

In vitro microdialysis with 30 kDa cut-off membranes (BR-2 probes, BASi) was also operated by a syringe pump. Recombinant α-synuclein from SensoLyte α-synuclein ELISA kit (Anaspec) was dissolved in aCSF at 1 μg/ml and prepared in an Axygen maximum recovery tube. During microdialysis, flow rate varied from 0.2–1.2 μl/min and α-synuclein concentration in each flow rate was determined by α-synuclein ELISA.

### Size exclusion chromatography

ISF samples were pooled and separated by a size exclusion chromatography using Superdex 75 column (GE healthcare). PBS was run at 0.5 ml/min with an AKTA explorer 10S (GE healthcare). α-Synuclein levels in each fraction were determined α-synuclein ELISA. LMW calibration kit to determine molecular weights was purchased from GE healthcare.

### α-Synuclein ELISA

α-Synuclein levels in media or dialysates were determined at the end of collection by SensoLyte α-synuclein ELISA kit (Anaspec).

### Lactate assay

Lactate levels in ISF were determined by lactate assay kit (Biovision).

### LDH assay

LDH activities were measured by cytotoxicity detection kit (Roche). A reference absorbance at 600 nm (A_600_) was measured and subtracted from absorbance at 490 nm (A_490_). Maximum LDH release (100%) was induced by adding 5% lysis buffer from a cytotoxicity detection kit to media and % cell toxicity was calculated.

### Statistical analysis

All statistical analysis was performed using Prism 6 (Graph Pad software). The comparison between baseline and treatment was done by two-tailed ratio paired t test. The comparison of two groups and multiple groups were done by two-tailed unpaired t test and one-way ANOVA with Tukey’s post-hoc test respectively.

### Animals

All animal studies were reviewed and approved by the Institutional Animal Care and Use Committee of the Graduate School of Medicine at the University of Tokyo. 2–6 month old wild type male mice on C57B6/J background were purchased from Japan SLC (Hamamatsu, Shizuoka, Japan).

## Abbreviations

GAPDH: Glyceraldehyde 3-phosphate dehydrogenase
ISF: Brain interstitial fluid
LDH: Lactate dehydrogenase
PTX: Picrotoxin
TTX: Tetrodotoxin
αLTX: α-latrotoxin
